# Clinicopathological Parameters Associated With Peritoneal Involvement in Epithelial Ovarian Tumors

**DOI:** 10.7759/cureus.36666

**Published:** 2023-03-25

**Authors:** Atif A Hashmi, Khushbakht Rashid, Muhammad Ghani Asif, Tanim Ud Dowlah, Abrahim H Ali, Umair Arshad Malik, Syed Munqaad Ali, Syed Jawwad Ali, Shamail Zia, Muhammad Irfan

**Affiliations:** 1 Pathology, Liaquat National Hospital and Medical College, Karachi, PAK; 2 Internal Medicine, Liaquat National Hospital and Medical College, Karachi, PAK; 3 Pathology, Multan Medical and Dental College, Multan, PAK; 4 Internal Medicine, Bangladesh Medical College, Dhaka, BGD; 5 Internal Medicine, Aga Khan University, Karachi, PAK; 6 Internal Medicine, Dow University of Health Sciences, Karachi, PAK; 7 Pathology, Dow University of Health Sciences, Karachi, PAK; 8 Pathology, Jinnah Sindh Medical University, Karachi, PAK; 9 Statistics, Liaquat National Hospital and Medical College, Karachi, PAK

**Keywords:** mucinous carcinoma, serous carcinoma, omental metastasis, ovarian tumors, peritoneal cytology

## Abstract

Introduction

Ovarian tumors remain one of the leading malignancies of the female genital tract, with a high mortality rate due to their insidious onset and lack of detection at an earlier stage. These tumors metastasize by direct extension into the neighboring pelvic organs; hence, the detection of peritoneal metastasis is valuable for staging and prognostic purposes. Peritoneal wash cytological analysis is an effective predictor of the involvement of the ovarian surface and peritoneal dissemination even in subclinical involvement of the peritoneum. The study aims to determine the significance of peritoneal wash cytology as a prognostic parameter and correlate it with various clinicohistological features.

Methods

A retrospective study was conducted at the Department of Histopathology, Liaquat National Hospital, Karachi, Pakistan, between July 2017 and June 2022. During this period, all the cases of ovarian tumors (borderline and malignant) that underwent total abdominal hysterectomy with bilateral salpingo-oophorectomy and omental and lymph node sampling were included in the study. After opening the abdominal cavity, the free fluid present was aspirated immediately, the peritoneum was lavaged with 50-100 mL of warm saline, and samples were collected and sent for cytological analysis. Four cytospin smear slides and cell block preparation were prepared. The findings of peritoneal cytology were correlated with various clinicohistological features.

Results

A total of 118 cases of ovarian tumors were included in the study. Serous carcinoma was the most common sub-type (50.8%), followed by endometrioid carcinoma (14.4%), and the mean age at diagnosis was found to be 49.9±14.9 years. The mean tumor size was 11.2 cm. The majority of the cases of ovarian carcinoma were of high grade (78.8%), with capsular invasion present in 61% of cases. Positive peritoneal cytology was noted in 58.5% of cases, with omental involvement in 52.5% of cases. Serous carcinoma showed the highest frequency of positive cytology (69.6%) and omental metastasis (74.2%). Apart from tumor type, positive peritoneal cytology showed a significantly positive correlation with age, tumor grade, and capsular invasion.

Conclusion

Based on our study findings, we conclude that peritoneal wash cytology is a sensitive indicator of the peritoneal spread of ovarian carcinoma, with a significant prognostic value. Serous carcinomas, especially high-grade with capsular invasion, were found to be predictors of peritoneal involvement of ovarian tumors. Although we found smaller tumors to be associated with peritoneal disease more compared to larger ones, this most likely is attributed to tumor histology, as larger tumors were most commonly mucinous compared to serous carcinomas.

## Introduction

Ovarian cancer (OC) remains one of the most common cancers of the female genital tract. It is the second most common gynecological cancer after cervical cancers with the highest mortality rate and is ranked fifth among gynecological malignancies causing malignancy-related deaths. According to a study conducted in 2020, approximately 314,000 new cases of OCs were reported globally [1]. OCs are a heterogeneous group of malignancies having different etiologies, histological features, and molecular biology, and are more prevalent in post-menopausal women over 65 years of age [2,3].

Based on the anatomical origin, ovarian tumors are classified into three major categories, namely, epithelial tumors, sex cord-stromal tumors, and germ cell tumors. Among these categories, around 90% of the primary malignant ovarian tumors are epithelial in origin, making it the most common type, accounting for approximately 60% of all ovarian tumors [4]. The ovarian epithelial tumors are further classified based on morphology into serous carcinomas, which are further classified into high-grade and low-grade serous carcinomas, mucinous carcinomas, clear cell carcinomas, endometrioid carcinomas, transitional cell tumors, and mixed and undifferentiated types. These tumors can be further subdivided into benign, malignant, and those that have intermediate features referred to as borderline malignant [5]. Among these, the most common subtype is high-grade serous ovarian carcinoma, accounting for 70-75% of cases of epithelial ovarian tumors [6]. OCs have a high mortality rate due to their aggressive nature. Failure of early detection of these tumors due to the insidious onset, lack of symptoms in early stages, and lack of screening results in the detection of these tumors at an advanced stage [7,8]. Approximately 75% of women are diagnosed at an advanced stage when the tumor has already metastasized within the peritoneal cavity. These tumors usually metastasize by direct extension into the neighboring pelvic organ or by seeding of the detached cancer cells onto the peritoneal structures via dissemination through peritoneal fluid. As the tumor advances locally, it encases the sigmoid colon and involves the omentum covering the bowel and the abdominal cavity, resulting in ascites and abdominal discomfort; rarely do these tumors metastasize through the vasculature [9]. The five-year survival rate of OC diagnosed at an advanced stage is only 29% [10].

The International Federation of Gynecology and Obstetrics (FIGO) provided staging of ovarian carcinoma, according to which positive peritoneal cytology is observed in stages IC/IIA/IIB or IIC [11]. Detection of peritoneal and omental metastasis in ovarian tumors is of great significance, as it is required for the staging of the disease and is associated with poor prognosis and increased incidence of recurrence [12]. Peritoneal wash cytology (PWC) analysis and omental sampling were performed during surgical procedures of suspected malignant ovarian tumors to detect the peritoneal and omental spread of the disease. The current study determined the frequency of the presence of peritoneal and omental disease in ovarian tumors by analyzing the PWC and omental sampling and correlating peritoneal cytology and omental metastasis with various prognostic clinicopathological features of ovarian tumors.

## Materials and methods

We conducted a retrospective, cross-sectional study at the Department of Histopathology, Liaquat National Hospital, Karachi, Pakistan. The biopsy-proven borderline and malignant ovarian tumor cases reported over five years from July 2017 to June 2022 were enrolled in the study. All the cases included underwent bilateral oophorectomy with total abdominal hysterectomy, and omental and pelvic lymph node sampling along with peritoneal cytology. Cases of benign ovarian tumors were excluded from the study.

At the time of laparotomy, after opening the abdominal cavity, any free fluid present in the abdomen was aspirated immediately after entry into the peritoneum; various areas of the peritoneum were instilled with 50-100 mL of warm normal saline, which was aspirated after 5-10 minutes. Similarly, omental tissue samples were collected. All the specimens collected during the surgery were sent to the histopathology lab for cytological and histopathological analysis.

The peritoneal fluid and ascitic fluid aspirates were centrifuged, and four cytospin smear slides were prepared, which were fixed with 95% alcohol. The material left was used for cell block preparation. The smear slides were stained with hematoxylin and eosin and Papanicolaou stains and were examined under a microscope by a senior histopathologist of Liaquat National Hospital.

The immunohistochemical stains including pan-cytokeratin, calretinin, and epithelial cell adhesion molecule (EPCAM/Ber-EP4) were performed on the cell block material of certain cases in which the confirmation of diagnosis was required.

The histopathologists interpreted the results of the peritoneal and ascitic fluid cytology without any prior knowledge of the surgical pathological findings. The peritoneal cytology and the presence of omental metastasis were labeled positive when the following criteria were met: the presence of an abnormal group of cells, chromatin and nucleolar abnormalities, disordered nuclear arrangement, and karyomegaly.

The results of peritoneal cytology and omental metastasis were correlated with the following parameters: the size of the tumor, age at diagnosis, type, and grade of ovarian tumor.

Data analysis was performed using the Statistical Package for Social Sciences (Version 26.0, IBM Inc., Armonk, NY, USA). Means were calculated for the size of the tumor and the age at diagnosis. Chi-square and Fisher’s exact tests were applied to determine the association of peritoneal cytology and omental metastasis with the prognostic parameters. A p-value of <0.05 was taken as significant.

## Results

A total of 118 cases of ovarian tumors were included in the study. The mean age at diagnosis was found to be 49.9±14.9 years. Women over 50 years of age (52.5%) had the highest frequency of ovarian tumors in our study. The mean size of the tumor was found to be 11.2 cm. Serous carcinoma appeared to be the most prevalent malignancy overall, occurring in 50.8% of cases, followed by endometrioid carcinoma (14.4% of cases) and mucinous carcinoma (13.6%). The study demonstrated that ovarian tumors in the majority of cases were of high grade (78.8%), with 61% of cases exhibiting capsular invasion. Only 14.4% of individuals had a low-grade tumor, and 39% of cases showed no evidence of capsular invasion. Omental metastases and peritoneal cytology were positive in 52.5% and 58.5% of cases, respectively (Table 1).

**Table 1:** Clinicopathological features of study population SD, standard deviation

Clinicopathological features	Values
Age (years)	
Mean±SD	49.99±14.91
Age groups	
≤35 years, n (%)	28 (23.7)
36-50 years, n (%)	28 (23.7)
>50 years, n (%)	62 (52.5)
Tumor size (cm)	
Mean± SD	11.25±7.978
Tumor size	
<10 cm, n (%)	65 (55.1)
10-20 cm, n (%)	37 (31.4)
>20 cm, n (%)	16 (13.6)
Tumor type	
Serous carcinoma, n (%)	60 (50.8)
Endometrioid carcinoma, n (%)	17 (14.4)
Mucinous carcinoma, n (%)	16 (13.6)
Clear cell carcinoma, n (%)	6 (5.1)
Carcinosarcoma, n (%)	5 (4.2)
Borderline serous tumor, n (%)	4 (3.4)
Borderline mucinous tumor, n (%)	10 (8.5)
Tumor grade	
Low grade, n (%)	17 (14.4)
High grade, n (%)	93 (78.8)
Not applicable, n (%)	8 (6.8)
Capsular invasion	
Present, n (%)	72 (61)
Absent, n (%)	46 (39)
Peritoneal cytology	
Positive, n (%)	69 (58.5)
Negative, n (%)	49 (41.5)
Omental metastasis	
Positive, n (%)	62 (52.5)
Negative, n (%)	56 (47.5)

Table 2 shows a significant association of positive peritoneal cytology with age, tumor size, tumor type, grade, and capsular invasion. Peritoneal cytology was positive mostly among women older than 50 years (58% of cases), followed by women between 36 and 50 years of age. Conversely, tumors of smaller size (<10 cm) were found to have a significantly higher incidence of positive peritoneal cytology (79.7%). The highest incidence of positive peritoneal cytology was found in serous carcinoma (69.6% of cases), followed by endometrioid carcinoma (10.1% of cases). High-grade tumors were found to have a higher frequency of positive peritoneal cytology (91.3% of cases) compared with low-grade tumors that showed positive cytology only in 5.8% cases. Similarly, 85.5% cases with capsular invasion showed positive peritoneal cytology compared with the cases with no evidence of capsular invasion, in which positive peritoneal cytology was found to be present in only 14.5% cases.

**Table 2:** Association of peritoneal cytology with clinicopathological features *Significant as p-value < 0.05

Clinicopathological features	Values		p-Value
	Peritoneal cytology		
	Positive	Negative	
Age groups			
≤35 years, n (%)	8 (11.6)	20 (40.8)	0.001*
36-50 years, n (%)	21 (30.4)	7 (14.3)	
>50 years, n (%)	40 (58)	22 (44.9)	
Tumor size			
<10 cm, n (%)	55 (79.7)	10 (20.4)	<0.001*
10-20 cm, n (%)	12 (17.4)	25 (51)	
>20 cm, n (%)	2 (2.9)	14 (28.6)	
Tumor type			
Serous carcinoma, n (%)	48 (69.6)	12 (24.5)	<0.001*
Endometrioid carcinoma, n (%)	7 (10.1)	10 (20.4)	
Mucinous carcinoma, n (%)	4 (5.8)	12 (24.5)	
Clear cell carcinoma, n (%)	4 (5.8)	2 (4.1)	
Carcinosarcoma, n (%)	4 (5.8)	1 (2)	
Borderline serous tumor, n (%)	1 (1.4)	3 (6.1)	
Borderline mucinous tumor, n (%)	1 (1.4)	9 (18.4)	
Tumor grade			
Low grade, n (%)	4 (5.8)	13 (26.5)	<0.001*
High grade, n (%)	63 (91.3)	30 (61.2)	
Not applicable, n (%)	2 (2.9)	6 (12.2)	
Capsular invasion			
Present, n (%)	59 (85.5)	13 (26.5)	<0.001*
Absent, n (%)	10 (14.5)	36 (73.5)	

Table 3 shows a significant association of omental metastasis with tumor size, tumor type, grade, and capsular invasion. Although the presence of omental metastasis was higher in patients diagnosed with ovarian tumor at >50 years of age, i.e., 61.3 % of women of >50 years of age had omental metastasis; however, the finding was not statistically significant. Conversely, the frequency of omental metastasis was higher with smaller tumor sizes, i.e., 71% of cases of tumor size < 10 cm had positive omental metastasis, which is significantly higher compared to other age groups. The tumor type having the highest frequency of omental metastasis is serous carcinoma (74.2%), and the incidence of omental metastasis was significantly low in other sub-types. The high-grade tumors had a significantly higher incidence of omental metastasis, (90.3% of cases), as compared to low-grade tumors that had omental metastasis (6.5% of cases). The capsular invasion was found to correlate positively with omental metastasis as 83.9% of cases with capsular invasion were found to have omental metastasis, while the incidence of omental metastasis was significantly low in tumors with no capsular invasion.

**Table 3:** Association of omental metastasis with clinicopathological features *Significant as p-value < 0.05

Clinicopathological features	Values		p-Value
	Omental metastasis		
	Positive	Negative	
Age groups			
≤35 years, n (%)	12 (19.4)	16 (28.6)	0.135
36-50 years, n (%)	12 (19.4)	16 (28.6)	
>50 years, n (%)	38 (61.3)	24 (42.9)	
Tumor size			
<10 cm, n (%)	44 (71)	21 (37.5)	0.001*
10-20 cm, n (%)	14 (22.6)	23 (41.1)	
>20 cm, n (%)	4 (6.5)	12 (21.4)	
Tumor type			
Serous carcinoma, n (%)	46 (74.2)	14 (25)	<0.001*
Endometrioid carcinoma, n (%)	4 (6.5)	13 (23.2)	
Mucinous carcinoma, n (%)	4 (6.5)	12 (21.4)	
Clear cell carcinoma, n (%)	4 (6.5)	2 (3.6)	
Carcinosarcoma, n (%)	2 (3.2)	3 (5.4)	
Borderline serous tumor, n (%)	1 (1.6)	3 (5.4)	
Borderline mucinous tumor, n (%)	1 (1.6)	9 (16.1)	
Tumor grade			
Low grade, n (%)	4 (6.5)	13 (23.2)	0.005*
High grade, n (%)	56 (90.3)	37 (66.1)	
Not applicable, n (%)	2 (3.2)	6 (10.7)	
Capsular invasion			
Present, n (%)	52 (83.9)	20 (35.7)	<0.001*
Absent, n (%)	10 (16.1)	36 (64.3)	

## Discussion

This study was conducted to determine the frequency of positive peritoneal cytology and omental metastasis in ovarian tumors and its association with various factors including age, tumor size, tumor histology, and grade. We found that positive peritoneal disease was found in a significant number of cases, signifying a late disease presentation in our setup. Moreover, a significant association of peritoneal disease was noted with serous histology, high grade, and capsular invasion.

Our study demonstrated that serous carcinoma (50.8%) was found to be the most prevalent type of ovarian carcinoma followed by endometrioid carcinoma (14.4%). We found that the majority (52.5%) of the women were over 50 years of age at the time of diagnosis. Similar to our study, a recent study showed that the incidence of OC is higher among women over 65 years of age [3], while according to some studies conducted previously, the median age at the time of diagnosis varies between 50 and 79 years [13-15].

Funt et al. conducted a study on ovarian malignancies, and similar to our study, they found serous carcinoma to be the most common epithelial ovarian carcinoma [16]. Similarly, Di Giorgio et al. conducted a study on 70 patients with advanced OC and found serous carcinoma as the most common type (71.5%), followed by mucinous carcinoma (17.3%) [17].

Ovarian tumors spread by direct extension into the pelvic structures or the malignant cells are shed into the peritoneal cavity that gets lodged into the peritoneal cavity; the most common sites of metastasis of OC are the peritoneum and omentum (86%), bowel (50%), and spleen (20%) [18]. Sehouli et al. conducted a study on the pattern of intra-abdominal tumor dissemination and surgical outcome of patients with primary OC. Similar to our study, they found that the average age at diagnosis was > 50 years (median age 57.7 years) and they reported that tumor dissemination was the most common in the peritoneum (76%), followed by the colon (52%) [19].

PWC is of great significance as it helps detect peritoneal dissemination; even sub-clinical peritoneal spread may be detected by PWC and hence provide valuable information regarding staging and prognosis [20]. To determine the sensitivity of PWC, two parameters are used: omental metastasis and peritoneal histology. In our study, positive peritoneal cytology was seen in 58.5% of cases, and the presence of omental metastasis was noted in 52.5% of cases. We also found that 78.8% of all cases were of high grade with capsular invasion present in 61% of cases. Various studies have been conducted to determine the detection rate of peritoneal spread by peritoneal cytology that varied with various studies. Gulzar et al. reported a cytology detection rate of 41.6%, Ozkara et al. reported a rate of 62.2%, and Sanches and Matsubara reported a rate of 20% [21-23].

Our study demonstrated that positive peritoneal cytology and omental metastasis were more common in women over 50 years of age as compared to women diagnosed with OC at a younger age; hence, we concluded that the incidence of positive peritoneal cytology had a positive correlation with older age. Our study also showed that the frequency of positive peritoneal cytology and omental metastasis was more with smaller tumors of <10 cm. This finding is likely due to the fact that most large-sized tumors were mucinous. Serous carcinoma was most frequently associated with positive peritoneal cytology (69.9%) and omental metastasis (74.2%), followed by endometrioid carcinoma. As borderline serous and mucinous tumors showed a significantly lower frequency of positive peritoneal cytology and omental metastasis, we concluded that serous carcinoma had a positive correlation with positive PWC.

Sneige et al. conducted a comparative study and analyzed PWC of serous carcinoma of low grade; they found that the detection rate of positive peritoneal cytology was 27% and also found 69% of patients with peritoneal implants to have serous carcinoma. They concluded that PWC is a sensitive predictor of peritoneal dissemination even in serous carcinoma of low grade [11]. Arora et al. proved that serous carcinoma diagnosed at an older age (> 60 years) was associated with poor prognosis [24]. Fadare et al. conducted a study and found that the positive peritoneal cytology rate in patients with OC was 25%, with serous carcinoma being the most frequent sub-type with positive cytology, and clear cell carcinoma being the least frequent type [25].

Tumors with ovarian capsular invasion grow exophytically, and malignant cells are shed into the peritoneal cavity that gets implanted in the peritoneal cavity, which eventually results in peritoneal dissemination [26]. Determination of capsular integrity is imperative as it upstages the tumor from stage 1 to stage 1C2 and stage 1C3 (positive cytology) [27]. Therefore, in our study, we tried to determine the association of capsular invasion with PWC. We found that the capsular invasion was associated with positive peritoneal cytology in 85.5% of cases and exhibited omental metastasis in 83.9% of cases; hence, capsular invasion seemed to have a statistically significant correlation with positive peritoneal cytology and omental metastasis.

Our study also demonstrated that high-grade tumors showed a significantly higher incidence of positive cytology (91.3%) and omental involvement (90.3%). A study was conducted in Pakistan by Gulzar et al. on the significance of PWC for accurate staging of OCs. They found positive peritoneal cytology to be correlated to tumor stage [21]. A study was conducted by Yoshimura et al., in which 90 patients with OCs were examined. Similar to our study, they found that serous and endometrioid carcinoma were associated with positive peritoneal cytology [28]. Similarly, another study found that serous carcinoma was associated with positive peritoneal cytology (76.9%) followed by endometrioid (44%) and mucinous carcinoma (25%), and a positive association was found between capsular invasion and positive peritoneal cytology and omental metastasis [29].

There were a few limitations of our study as this was a retrospective study and patient’s follow-up was not determined. Moreover, this was a single-center study with a limited sample size, and molecular and biomarker profiles were not evaluated. Therefore, we suggest further prospective multi-center studies in our population to accurately determine prognostic parameters (both histological and molecular) of OC in our setup.

## Conclusions

We concluded that ovarian tumors were most prevalent among women over 50 years of age in our setup. Serous and endometrioid carcinomas were found to be the most prevalent among our subjects. The factors associated with an adverse prognosis, such as positive peritoneal cytology and omental metastasis, were found to be more frequently positive in high-grade serous carcinoma, followed by endometrioid carcinomas. Larger tumors showed less frequency of peritoneal involvement, but that is because mucinous tumors are generally large compared with serous tumors. Moreover, positive peritoneal cytology was associated with capsular invasion. Peritoneal cytology and omental metastasis are important prognostic factors for ovarian tumors. Therefore, PWC and omental sampling should be performed routinely in the surgical management of ovarian tumors to detect subclinical metastasis.
